# Conservative versus operative treatment of FFP II fractures in a geriatric cohort: a prospective randomized pilot trial

**DOI:** 10.1038/s41598-023-43249-w

**Published:** 2023-09-26

**Authors:** Darius M. Thiesen, Gerrit Althoff, André Strahl, Tim Rolvien, Karl-Heinz Frosch, Leon-Gordian Koepke, Christian Arras, Tobias M. Ballhause, Dimitris Dalos, Maximilian J. Hartel

**Affiliations:** 1 Department of Trauma and Orthopaedic Surgery, University Medical Center Hamburg-Eppendorf, Martinistr. 52, 20246 Hamburg, Germany; 2 Department of Trauma, Orthopaedic Surgery, and Sports Traumatology, BG Trauma Hospital Hamburg, Hamburg, Germany; 3 UKE Athleticum, Center for Athletic Medicine, University Medical Center Hamburg-Eppendorf, Hamburg, Germany

**Keywords:** Trauma, Musculoskeletal system, Bone

## Abstract

A clear recommendation regarding treatment strategy of fragility fractures of the pelvic ring is missing. The most typical fracture pattern is a lateral compression type injury with non-displaced fractures of the anterior pubic rami and a unilateral os sacrum fracture (FFP II). We hypothesized that operative treatment would be superior to conservative treatment. From October 2017 to April 2020, a randomized prospective non-blinded trial with n = 39 patients was carried out. Two arms with 17 operative versus 22 conservative cases were created. Inclusion criteria were a posterior pelvic ring fracture FFP type II, age over 60 years and acute fracture (< 3 weeks). Barthel index, pain level (VAS), quality of life (EQ-5D-3L), and Tinetti–Gait Test were determined on admission, at discharge, and after 3, 6, 12 and 24 months. Median follow-up was 12.9 months. The Barthel index (= 0.325), VAS (p = 0.711), quality of life (p = 0.824), and Tinetti–Gait Test (p = 0.913) showed no significant differences between the two groups after 12 months. Two patients switched from the conservative to the operative arm due to persistent immobilization and pain. The one-year mortality rate showed no significant difference (p = 0.175). Our hypothesis that surgical treatment is superior was refuted. No significant benefit was shown in terms of quality of life, mortality and pain levels. The results suggest a more differentiated treatment approach in the future, with initial conservative treatment preferred. A larger multi-center trial is required to confirm these findings.

**Trial registration:** The study was retrospectively registered with the German Clinical Trials Registry (DRKS00013703) on 10/12/2018.

## Introduction

The incidence of low-energy pelvic ring fractures has sharply increased over the past decade and is projected to continue doing so^1,2^. The expected increase will mainly be driven by an aging population and a more stringent diagnostic algorithm for low back pain in older patients^3,4^. Choosing between conservative versus operative treatment is a decision that orthopedic surgeons will often have to face. In contrast to high-velocity pelvic fractures in young individuals, geriatric fractures show a dynamic fracture development, sometimes without an explicit trigger^5^. Fracture dislocation may continue, beginning with a unilateral fracture of the pubic ramus which then possibly progresses to spinopelvic dissociation. Because of this unique behavior of fragility fractures, new classification systems have been developed^6^. In this context, the most common insufficiency fracture was a fracture of the anterior pubic branch and unilateral os sacrum without major dislocation, accounting for approximately 51% of cases^6^. This would classify as a type II FFP injury according to the Fragility Fracture of the Pelvis (FFP) classification^6^.

Despite the increasing incidence of fragility fractures of the pelvis, adequate and evidence based treatment strategies are still missing. For high-energy trauma resulting in unstable and dislocated pelvic fractures, surgical stabilization is known to reduce mortality^7,8^. For pelvic ring fractures in the elderly, a United States nationwide data analysis found a lower mortality in patients receiving operative treatment^1^. Two retrospective cohort studies reported inconclusive results; Hoech et al.^9^ showed a higher 2-year mortality in patients treated conservatively, whereas Schmitz et al.^10^ found no difference in mortality but reported more complications in patients treated surgically. To our knowledge, existing studies demonstrate a selection bias regarding surgical therapy in this frail cohort. To overcome this problem, a single-center prospective randomized controlled study was conducted. We hypothesized that operative treatment would be superior to conservative treatment.

## Materials and methods

This randomized prospective nonblinded study (PRESS—Prospective Randomized Evaluation of Sacral fractureS), was conducted from October 2017 to April 2020 with a parallel design at the University Medical Center Hamburg-Eppendorf. The mid-term outcomes of two different treatment options in elderly patients with sacral fractures were evaluated. Patients older than 60 years with a type B2.1 or FFP II posterior pelvic ring fracture and a fracture age of less than 3 weeks were eligible for inclusion. Patients under 60 years of age also met the inclusion criteria if they had a previously confirmed history of osteoporosis.

Exclusion criteria were metastatic tumors in the pelvis, pathologic fractures, or a high-energy trauma mechanism. The diagnosis was confirmed using CT imaging. Appropriate informed consent was obtained from the patient’s or their legal guardians. The participants were randomized using a previously prepared randomization list with a random sequence of zeros and ones representing the two treatment arms of the study by a staff member at our hospital who was not involved in the study. Treatment group I received surgical treatment during the first three days after admission (ST group), while treatment group II received a comprehensive conservative treatment regime (CCT group). Data collection was planned at admission (t_0_), after 6 weeks (t_1_), 6 months (t_2_), 12 months (t_3_), and 24 months (t_4_). Recruitment and follow-up was discontinued due to ethical considerations once the first wave of the COVID-19 pandemic emerged and Germany went into lockdown in April 2020. The investigation team decided against seeing elderly patients without significant complaints in the outpatient clinic for their safety in a general lockdown situation. Therefore, data are available up to measurement time t_3_.

### Treatment

**Figure 1:**
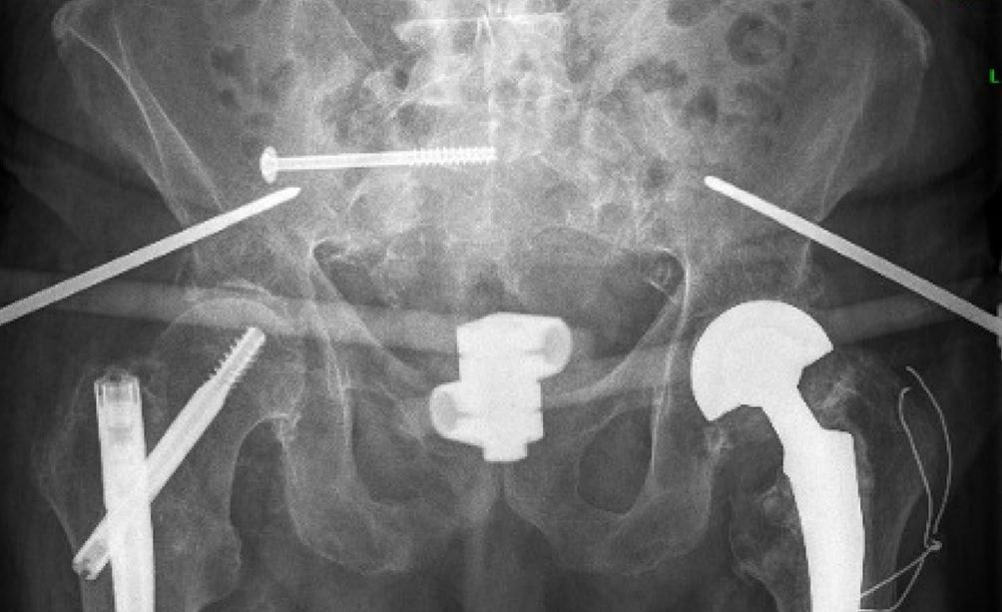
Showing an X-ray postoperatively after percutaneous S1 Screw fixation and application of anterior supraacetabular external fixator.

In the ST group, percutaneous minimally invasive navigated S1 sacroiliac screw osteosynthesis (^®^Depuy Synthes canulated 7.3 mm, partially threaded screws) was performed, with additional stabilization of the anterior pelvic ring using a supraacetabular external fixator (^®^Depuy Synthes AO external fixator) within the first 3 days after admission (Fig. 1). Both groups received analgesia according to the World Health Organization pain medication regimen, with the maximum daily dose of 4 × 1 g of Metamizole and additionally 2 × 5–10 mg of long-acting Oxycodone adapted to bodyweight. Every patient received postoperative pelvic radiographs. Early mobilization with full weight-bearing was encouraged with guided individual physical therapy exercises in all patients. Daily assessments were conducted on the CCT cohort to monitor the progression of pain and mobility. A patient was categorized as ambulatory if they could independently traverse 20 m within the ward while being assisted by a high walker.

In the CCT group radiological follow-up imaging was performed after 3–5 days to exclude significant secondary dislocation. If mobilization was impossible or if severe pain (VAS > 5) still occurred during mobilization after 3 days, CCT participants changed study arms and were scheduled for surgery. These specific patients were assigned to the ST group in the statistical evaluation.

### Outcome measures

Data were collected at admission by Barthel questionnaires^11^ as an indicator of skills of daily living regarding independence or need for care. Visual analogue scale (VAS) was used to measure pain intensity. Life quality was assessed by the EQ-5D-3L^12^ questionnaire and the Tinetti–Gait Test^13^ was performed to measure static and dynamic balance. The occurring deaths were determined by contacting or receiving messages from the patient’s relatives. In addition, the German Pension Fund was contacted to receive the times of death.

### Statistical analysis

SPSS statistical program 25.0 (SPSS, Chicago, IL) and GraphPad Prism 9 (GraphPad Software, La Jolla, CA) were used for statistical analyses. Continuous variables are expressed as mean ± standard deviations (SD), while categorial variables are expressed as numbers and percentages. Normality distribution of the data was analyzed using the Shapiro–Wilk test.

For normally distributed data, a Bonferroni-adjusted Student’s t-test assessed possible differences between the ST and CCT groups at each of the measurements and the Mann–Whitney-U-Test for non-normal distributed data. Longitudinal analyses were performed as per protocol (PP) and intention-to-treat (ITT) analyses using the conservative last observation carried forward (LOCF) method in case of missing data for non-deceased patients. Survival rate was estimated by the Kaplan–Meier method with univariate Log-Rank test used to compare the ST group with the CCT group. In accordance with accepted standards, statistical significance was set to a 2-tailed p-value of 0.05.

### Sample size calculation

The sample size was calculated for the primary outcome, mortality at 24 months follow-up, with the scope of non-inferiority of the non-surgical. This calculation was performed considering the expected effect size, power, and design effect (log rank test). Based on a crossover rate of 11% reported by Höch et al.^9^, a conclusive sample of 130 patients was required to calculate significant differences.

### Ethical approval

This study conforms to the Declaration of Helsinki, was approved by the local research ethics committee called “Ärztekammer Hamburg” (reference number: PV5550) and was retrospectively registered with the German Clinical Trials Registry (DRKS00013703) on 10/12/2018.

### Consent to participate

For this vulnerable group, written informed consent was obtained from all participants or their legal representatives. If no legal guardian was available but the patient was mentally impaired (dementia), a legal guardian was appointed by the Hamburg authority in an expedited procedure after a personal assessment by a local judge within a few days.

### Award

This trial received the award as best evidence-based Study provided by the German Society for Orthopedic and Trauma Surgery (DGOU) in 2021.

## Results

**Figure 2:**
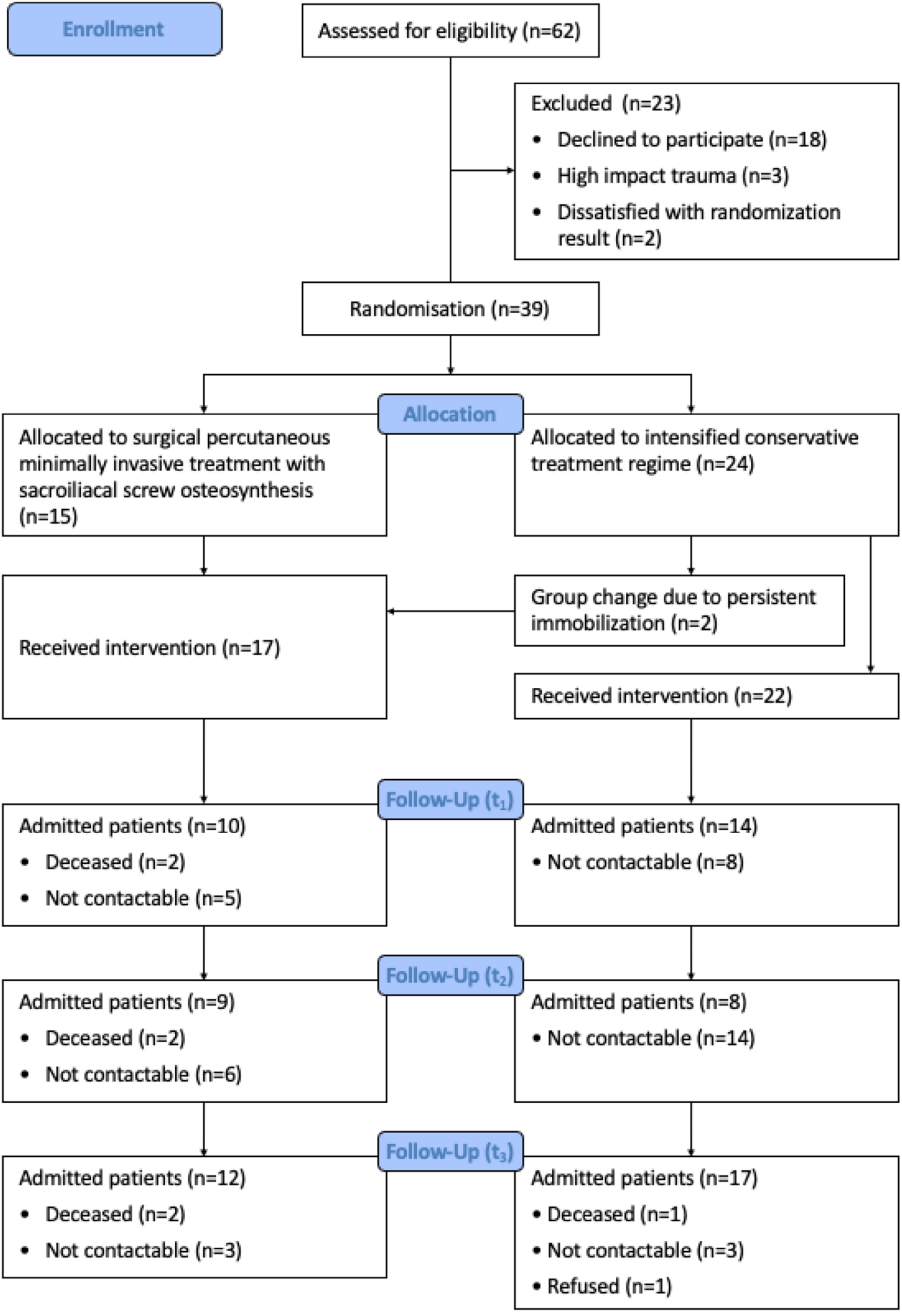
Flow chart of participants in the study according to CONSORT statement guidelines.

Between October 2017 and April 2020, the study enrolled 39 participants (92.3% female/7.7% male), out of a total of 62 possible patients with non- or minimally displaced geriatric sacral fractures. Two patients rejected study participation because they disagreed with the treatment option randomized to them and 18 cases refused to participate because they rejected the general randomization. Three patients were excluded due to a high impact trauma mechanism. Surgery was performed in 15 cases. Twenty-four patients were assigned to the CCT group. Two of them transferred into the ST group. Both transfers were due to persistent and immobilizing pain (Fig. 2).

The mean age was slightly higher in the CCT group (83.7 ± 8.9 years) compared to the ST group (77.5 ± 11.0). This difference was not significant (*p* = 0.06). There were no significant baseline differences between groups in terms of sex, pain, Barthel-index, Tinetti–Gait Test, quality of life and the mean number of comorbid conditions, see also Table 1. Single comorbidities were not clustered in one group, too. The predominant fracture subtype observed was FFP IIB, encompassing 30 patients, while FFP IIC was identified in 8 patients, and FFP IIA fracture was observed in one patient. No statistically significant variance in distribution between the Comprehensive Conservative Treatment (CCT) and Surgical Treatment (ST) groups was evident, as indicated in Table 2.

**Table 1:** Baseline characteristic of the surgical therapy and non-surgical treatment group.

Variable	Surgical	Non-surgical	*p*-value
Sex, M:F	3:14	0:22	0.074
Mean age, years (SD)	77.5 (11.0)	83.7 (8.9)	0.058
Mean number of comorbid secondary diagnoses (SD)	3.3 (1.6)	4.3 (2.0)	0.094
Mean VAS-pain (SD)	69.9 (27.3)	69.0 (22.2)	0.640
Mean Tinnetti-test score (SD)^a^	13.6 (10.5)	17.9 (8.6)	0.280
Mean Barthel-Index (SD)	90.3 (10.2)	85.5 (18.4)	0.715
Mean EQ-5D-3L index value (SD)^a^	0.69 (0.26)	0.62 (0.32)	0.712

^a^Measured after 6 weeks.

**Table 2:** Shows the fracture distribution in the two groups.

Type of fracture according to classification	Number of patients	ST-group (Surgical)	CCT-group (non-surgical)	p-value
FFP II A	1	0	1	–
FFP II B	30	18	12	0.77
FFP II C	8	5	3	0.89

### Complications and survival rate

**Figure 3:**
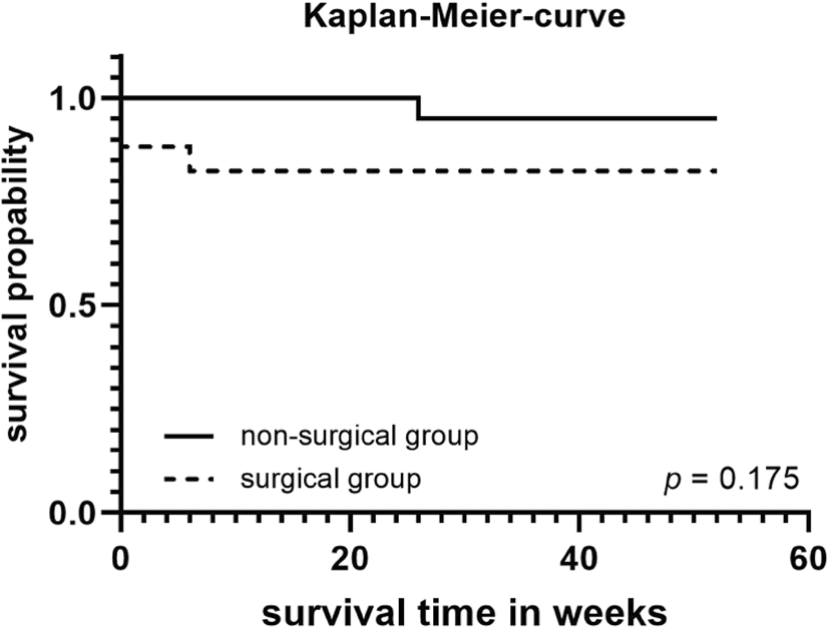
1-year Kaplan–Meier-curve patient survival compared between patients with surgical and non-surgical treatment.

The median follow-up time was 12.9 months. Ten patients (25.6%) were lost to follow-up at 12 months. Three patients in the ST group and in the one patient in the CCT group died. Patients treated surgically showed a 17.6% complication rate (n = 3). One patient was diagnosed with a pin infection and loosening of the external fixator needing removal of the fixator. In another patient, asymptomatic loosening of the SI screw was noted, without the need for revision. A third patient had an infection of the surgical site due to inadequate wound care at the nursing home. There was no screw or pin misplacement noted in the cohort. The overall patient survival probability rates were 92.3% at 6 weeks and 89.5% at 6 and 12 months. The CCT group survival rates were 100% at 6 weeks and 95.0% at 6 and 12 months. Patients who were treated surgically showed a survival rate of 82.4% at 6 weeks as well as at 6 and 12 months. Nevertheless, no significant difference between the ST and CCT group survival rates was observed (Log Rank Chi^2^ = 1.842, *p* = 0.2; Fig. 3). The two patients who switched between treatment cohorts were encompassed within the ST group for all subsequent assessments of outcomes. Despite conducting computations with these two individuals excluded, no statistically noteworthy distinctions were observed between the groups.

### Intention-to-treat and per protocol outcome analyses

**Figure 4:**
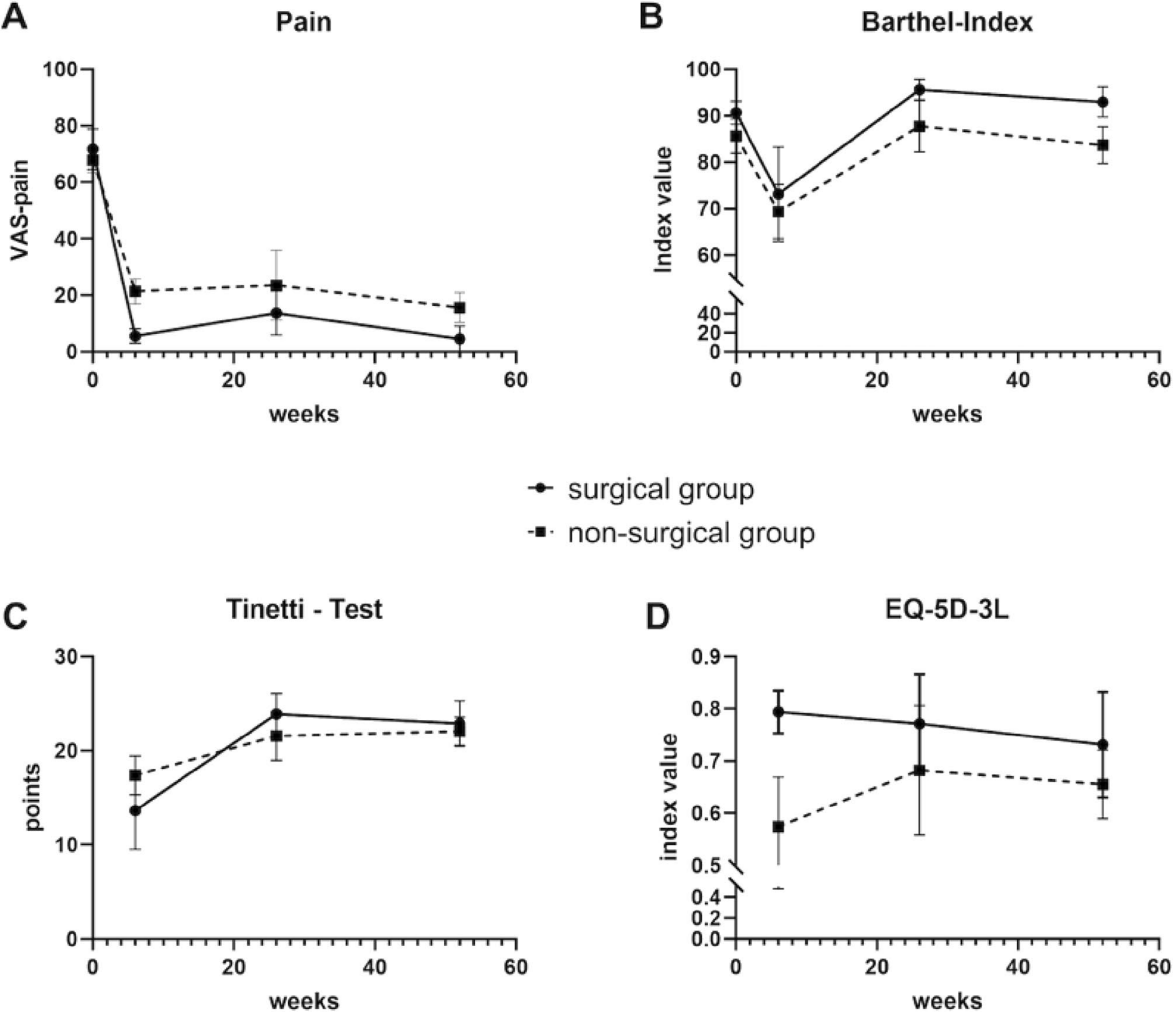
Short- and mid-term improvements of primary outcome measures.

When comparing the preoperative to the follow-up measurements in all patients in the ITT multivariate analysis, there was a significant decrease in VAS-pain (*p* < 0.001, *W* = 0.349) and the Barthel index (*p* < 0.001, *W* = 0.768). The postoperative course indicates no significant change in EQ-5D-3L index scores (*p* = 0.574) over time and only a minor decreasing trend of the Tinetti–Gait Test score (*p* = 0.092). Group allocation had no significant effect on the change of outcome in all cases (group × time effect: *p* > 0.05), indicating that the outcome is similar in both groups. After 12 months, there were no significant differences in the Barthel index (*p* = 0.3), VAS pain (*p* = 0.7), quality of life (*p* = 0.8), and Tinetti–Gait Test (*p* = 0.9). For the primary outcomes, the PP analyses revealed similar results to the ITT evaluation: VAS pain (*p* = 0.01, *W* = 0.240), Barthel index (*p* = 0.05, *W* = 0.685), Tinetti–Gait Test (*p* = 0.3), EQ-5D-3L (*p* = 0.4). Again, group allocation had no significant effect on change of outcome (group × time effect: *p* > 0.05) and no differences were observed between ST and CCT groups 12 months after treatment (*p* > 0.05; Fig. 4A–D).

## Discussion

The aim of this study was to compare clinical outcomes as well as the one-year mortality of comprehensive conservative versus surgical treatment of low-energy posterior pelvic ring fractures that are typically found in older populations. We found no significant difference between the surgically and conservatively treated groups in need of care (Barthel Index), level of pain (VAS), quality of life (EQ5D), gait performance (Tinetti) and in terms of mortality.

Our data showed that non- to minimally displaced fractures of the posterior pelvic ring have a major impact on the patient’s well-being, which is reflected in all measured scores. However, pain improvement occurred quickly, usually within the first 6 weeks. It was achieved slightly faster in the surgical group and remained generally low after one year. The restoration of mechanical stability of the posterior pelvic ring using surgical stabilization is a well-established treatment for unstable pelvic fractures caused by high-velocity trauma^1,7^. In the elderly population, minimally invasive techniques have provided good results in low and “no energy” trauma cases, showing rapid pain relief and promoting early mobility^14–16^. However, some literature also indicates that there is no difference in pain and morphine consumption between surgical and non-surgical treatments^9,17^, which was confirmed by our data.

Rommens et al.^5^ report a dynamic evolution of fragility fractures in 14.2% of their patients, starting with a low-energy fall or even no-trauma event which results in a fracture of the pubic ramus that continues to the posterior pelvic ring and may progress to the contralateral side if left untreated^5,6^. This subgroup of patients, with a fracture that is not initially displaced on computed tomography and is therefore supposedly stable, suffers from persistent pain and immobilization. Hotta et al.^18^ reported 8.8% of their cases had fracture progression, whereas Ueda et al.^19^ found 22.8%, and Hoech et al.^9^ 18%. To date, scientific literature investigating how to reliably identify patients at risk for fracture progression is still missing. In our study, 2 out of 24 cases in the conservative arm (8%) switched to the surgical group after failure of conservative therapy and then improved adequately after screw stabilization. One patient had a FFP II b and one FFP IIc fractures type. However, we did not find any fracture progression from FFP II to a higher degree of dislocation in the rest of the conservatively treated cohort. This could be attributed to the fact that our conservative treatment was stopped as soon as mobilization failed after three days.

Successful surgical fixation via a transiliosacral bar, sacroiliac screw, or a triangular osteosynthesis of the posterior pelvic ring has already been described and validated in this particular cohort by the literature in recent years^9,15,20^. However, typical complications, like screw malposition, did not occur in our cohort. Complications due to osteoporotic bone, such as screw or external fixator pin loosening, have been reported to be as high as 14%^21^. We found a similar rate in our study, with a total of 3 complications in 17 patients (18%). In one case (6%), we had a screw loosening of a posterior screw. This finding could be managed non-operatively due to missing soft tissue irritation and is similar to reported screw loosening rates of 2–10%^9,10^. In another case, a late, deep infection (4 weeks postoperatively) around the posterior screw was noted due to neglected aftercare in a nursing home. This required revision surgery with hardware removal and local debridement. Pin loosening due to superficial infection occurred in 1 out of 17 cases (6%).

The utilization of external fixation for stabilizing the anterior pelvic ring has become an established practice in the management of high-velocity trauma, particularly in cases involving open book fractures (Tile B1 or APC I) in young adults, as part of damage control surgery^7^. Previous studies have demonstrated its effectiveness in alleviating pain associated with pubic ramus fractures, but it also comes with inherent drawbacks, including the potential for pin loosening^22^. Notably, in a cohort of patients with osteoporotic bone, as observed in our study, the risk of pin failure becomes evident, affecting 2 out of 17 patients (12%).

Our primary objective was to pursue a minimally invasive approach, but it is essential to acknowledge that alternative techniques for stabilizing the anterior pelvic ring exist. These include open reduction and internal fixation using contoured plates or minimally invasive retrograde transpubic screw fixation that show adequate pain relief^23,24^.

The Barthel index was lower in the surgical patients in the first 6 weeks, which may be explained by the external fixator interfering with upright sitting and the daily pin care. However, the patient quality of life with an external fixation device was not found to be worse when compared to the conservative group.

The primary endpoint of our study was patient mortality during the 12-month follow-up period. An overall mortality of 4 out of 39 patients (10%) was measured, with three patients dying in the ST group, and one in the CCT group. It is known that immobilization, even for only 24 to 48 h, is one of the main risk factors for death in frail patients due to medical complications after a proximal femur fracture^25,26^. Similarly, we believe, avoiding immobilization is one of the cornerstones in the treatment of fragility fractures of the pelvic ring.

There are retrospective studies from the early 2000s without adequate diagnostics that investigated unilateral pubic ramus fractures in patients immobilized and hospitalized due to pain^27–30^. It must be assumed that a significant proportion of these patients also had an additional posterior ring fracture. Under this assumption, the observed therapy-independent 10–19% 1-year mortality described in these publications confirm our results. In a recent retrospective study, Hoech et al.^9^ reported a 30% mortality after two years in patients with an OTA B2.1 and B3.3 fracture (corresponding to a FFP II fracture), regardless of the treatment method. In their study, the conservative group had a 2-year mortality rate of 41%. The surgical and conservative groups had the same ASA score, but conservatively treated patients were significantly older. Randomization in our study prevented such this bias and our results showed no significant difference in mortality after 1 year. Our ST group had a non-significantly higher mortality rate of 17% when compared to 5% in the CCT group.

There are several limitations to our study. First, the number of patients included is not as high as was deemed necessary in our a priori analysis (n = 130), mainly due to the study participants not willing or able to be randomized and the COVID-19 pandemic, which forced us to discontinue recruitment and follow-up. This must be considered when interpreting the results of this randomized controlled trial, as its design is underpowered. However, this study represents the first attempt to prospectively investigate FFP II fractures. As usual when studying frail patient groups recruitment is difficult. Therefore, the information provided in this paper may likely add scientific information to the field of pelvic surgery, although it is underpowered. Secondly, we had a 25% loss of follow-up, which is not unusual in geriatric cohorts. Nevertheless, the achieved follow-up rate is still representative, especially in this older population. Thirdly, we conducted a non-blinded follow-up and evaluation because the nature of the surgery did not allow for blinded follow-up. Understandably, sham surgeries would have been unethical in a cohort of elderly with a number of pre-existing conditions. There are also some noteworthy strengths of our study. Most prominently, this is the first study to examine this emerging injury in a prospective manner without the surgical selection bias often seen in previous publications.

## Conclusion

The result of this randomized controlled pilot trial indicates that surgical treatment of FFP II fractures in geriatric patients may not be superior to conservative treatment. Non-displaced posterior pelvic ring fractures (FFP II) in an older population may be treated with comprehensive conservative therapy (analgesia and physical therapy with full weight bearing). It is crucial to note that if early mobilization is not possible, a switch to operative treatment should occur promptly, rather than attempting conservative management for an extended period. Vigilant follow-up is essential to prevent prolonged immobilization and detect any fracture progression.

The methodology used in this pilot trial may be valuable for designing larger, multi-center trials to confirm these findings and improve clinical decision-making for FFP II fractures in the elderly.

### Supplementary Information

Supplementary Information 1.

Supplementary Information 2.

## Data Availability

The datasets used and/or analyzed during the current study available from the corresponding author on reasonable request.
