# Supplementary material for: Conservative versus operative treatment of FFP II fractures in a geriatric cohort: a prospective randomized pilot trial
Source: Sci Rep. 2023 Sep 26;13:16124. doi: 10.1038/s41598-023-43249-w (PMC10522702; doi:10.1038/s41598-023-43249-w)
Supplement: Supplementary file 2 — Supplementary Information 2. [file 41598_2023_43249_MOESM2_ESM.docx]

**Studienprotokoll**

| 1. **Titel des Projektes:** |
| --- |
| **Prospektive Evaluation der operativen Versorgung von Sakrumfrakturen beim alten Menschen** |
| 1. **Wissenschaftliche Aufgaben:** |
| *Problemstellung und Stand des Wissens (mit Literaturangaben):* |
| Aktuell gibt es in der wissenschaftlichen Gemeinschaft kein klares Behandlungsregime für Sakrumfrakturen auf dem Boden einer Osteoporose des älteren Menschen des Typs B nach AO. Ferner sehen wir eine stark steigende Inzidenz dieser Fraktur-Entität^1^. Häufig schließt sich, besonders bei immobilisierenden Schmerzen und dem Nachweis einer vorderen/hinteren Typ B Beckenringfraktur eine minimalinvasive operative Therapie, zur Stabilisation der Verletzung an. Bei einem Blick in die Literatur fällt das Fehlen ausreichender Evidenz für das international weitgehend etablierte therapeutische Vorgehen auf. Dieses Vorgehen stützt sich weitestgehend auf Expertenmeinungen und Erfahrungsberichte aus Fallserien^2–6^  Die Fraktur des hinteren Beckenrings ist eine bedeutende Verletzung des alten Menschen. Sie entsteht zumeist durch Niedrig-Energie Traumen basierend auf eine verminderte Knochenqualität im Sinne einer Osteoporose^3,7^. Zunehmend treten auch Frakturen ohne Trauma, ausgelöst durch repetitive Kraftübertragung, beispielsweise beim Gehen^8^, des osteoporotisch veränderten Knochens in den Vordergrund.  Die gegenwärtige Literatur bietet keine eindeutige Behandlungsempfehlung.  In einem Review Paper von Rommens und Wagner et al. aus 2015 ^2^ werden auf Basis klinischer Erfahrung und anhand einer retrospektiven Studie Behandlungsempfehlungen bezüglich der Insuffizienzfrakturen (hier FFP = fragility fractures of the pelvis genannt) präsentiert. Die Autoren empfehlen zunächst jeden Patienten mit einer einseitigen Fraktur des hinteren Beckenrings ohne Dislokation und mit oder ohne Fraktur des ipsilateralen vorderen Beckenrings unter Analgesie zu mobilisieren. Wenn dies nicht gelänge, sei eine operative Therapie mittels Schraubenosteosynthese durchzuführen. Konträr dazu veröffentlichten die gleichen Autoren zwei Jahre zuvor eine Publikation^6^, in der sie nicht nur ein neues Klassifikationssystem (FFP) einführten, hier wurde die Empfehlung ausgesprochen zunächst alle, auch nicht dislozierten Frakturen des hinteren Beckenrings (FFP IIa-c) einer operativen Therapie zu unterziehen. Dies wurde unter der Prämisse konstatiert eine sekundäre Frakturdislokation unter Mobilisation zu verhindern. Leider bieten beide Veröffentlichungen lediglich Handlungsempfehlung mit dem, nach der deutschen Gesellschaft für Evidenzbasierte Medizin, Evidenzlevel III bis IV.  Betrachtet man die Datenlage bezüglich der konservativen Therapie ist eine Studie aus 2011 von Mears et al^4^ aufzuführen. Es wurden 181 Patienten über 65 Jahre nach Niedrig-Energie-Trauma mit einer vorderen und/oder hinteren Insuffizienzfraktur im Schnitt für 3,5 +/- 2,5 Jahre betreut. Insgesamt boten nur 14 Patienten eine Insuffizienzfraktur des Os sacrum, allerdings ist davon auszugehen, dass Patienten mit dislozierter ein- oder beidseitiger vorderer Beckenringfraktur zu mindestens 83-96,3% ^1,5^ ebenfalls eine nicht erkannte Fraktur des hinteren Beckenrings aufwiesen.  Die Therapie bestand aus Analgesie und Physiotherapie. Zusammenfassend stellen die Autoren fest, dass vordere und hintere Beckenringfrakturen einen großen Einfluss auf die eigenständige Mobilität und das unabhängige Leben der älteren Patienten haben. 30% der Patienten verloren die Fähigkeit sich eigenständig zuhause zu versorgen, die Zahl der Patienten, die nur noch in einem Pflegeheim versorgt werden konnten stieg nach Fraktur um 170% an. Insgesamt berichten die Autoren von einer posttraumatischen Mortalitätsrate von 23% nach einem und 43% nach 3 Jahren.  In anderen retrospektiven Studien, welche sich mit der konservativen Therapie beschäftigten, zeigten sich ebenfalls deutliche Einschnitte bei der Mobilität, Grad der Selbstständigkeit sowie Mortalitätsraten von 14 bis 18%^9,10^.  Studien zum Outcome nach operativer Versorgung mit transiliosakralen Schrauben im Sinne von Funktion, Schmerz und Lebensqualität gibt es zum aktuellen Zeitpunkt für jüngere Patienten mit guten Ergebnissen^11^. Bei Patienten älter als 60 Jahre mit Sakrumfraktur und operativer Versorgung durch eine transiliosakrale Schraubenosteosynthese fehlt eine wissenschaftliche Auseinandersetzung mit beispielsweise einem Follow-Up von mindestens 12 Monate und einer Kontrollgruppe. Eine Studie beschreibt Schmerzen unmittelbar vor Entlassung ohne Nachuntersuchung und Kontrollgruppe^12^, hier nahmen die Schmerzen nach operativer Therapie signifikant ab und eine Mobilisation war schneller möglich.  Zusammenfassend mangelt es der wissenschaftlichen Gemeinschaft an einem Konzept zur Therapie dieser wachsenden Herausforderung. Im angelsächsischen Raum wird weiterhin ein eher konservatives Therapieregime favorisiert, welches aus unserer Sicht in puncto Mortalität, Verschlechterung der Mobilität und Komorbidität eine unzureichende Behandlung darstellt. Studien zur operativen Therapie mit gutem Evidenzgrad (I-II) sind gegenwärtig nicht verfügbar, dennoch weisen einzelne Veröffentlichungen mit aussichtsreichen Ergebnissen ^2,12,13^ auf eine Verbesserung der Mobilität und Morbidität durch die operative Therapie hin. |
| Literatur:  1. Nüchtern, J. V *et al.* Significance of clinical examination, CT and MRI scan in the diagnosis of posterior pelvic ring fractures. *Injury* **46,** 315–9 (2015).  2. Wagner, D., Ossendorf, C., Gruszka, D., Hofmann, A. & Rommens, P. M. Fragility fractures of the sacrum: how to identify and when to treat surgically? *Eur. J. Trauma Emerg. Surg.* **41,** 349–62 (2015).  3. van den Bergh, J. P., van Geel, T. A. & Geusens, P. P. Osteoporosis, frailty and fracture: implications for case finding and therapy. *Nat. Rev. Rheumatol.* **8,** 163–172 (2012).  4. Mears, S. C. & Berry, D. J. Outcomes of displaced and nondisplaced pelvic and sacral fractures in elderly adults. *J. Am. Geriatr. Soc.* **59,** 1309–12 (2011).  5. Scheyerer, M. J. *et al.* Detection of posterior pelvic injuries in fractures of the pubic rami. *Injury* **43,** 1326–9 (2012).  6. Rommens, P. M. & Hofmann, A. Comprehensive classification of fragility fractures of the pelvic ring: Recommendations for surgical treatment. *Injury* **44,** 1733–44 (2013).  7. Dodge, G. *et al.* Low-impact pelvic fractures in the emergency department. *CJEM* **12,** 509–513 (2010).  8. Linstrom, N. J. *et al.* Anatomical and Biomechanical Analyses of the Unique and Consistent Locations of Sacral Insufficiency Fractures. *Spine (Phila. Pa. 1976).* **34,** 309–315 (2009).  9. Hill, R. M., Robinson, C. M. & Keating, J. F. Fractures of the pubic rami. Epidemiology and five-year survival. *J. Bone Joint Surg. Br.* **83,** 1141–4 (2001).  10. Taillandier, J., Langue, F., Alemanni, M. & Taillandier-Heriche, E. Mortality and functional outcomes of pelvic insufficiency fractures in older patients. *Joint. Bone. Spine* **70,** 287–9 (2003).  11. Khaled, S. A., Soliman, O. & Wahed, M. A. Functional outcome of unstable pelvic ring injuries after iliosacral screw fixation: single versus two screw fixation. *Eur. J. Trauma Emerg. Surg.* **41,** 387–392 (2015).  12. Hopf, J. C., Krieglstein, C. F., Müller, L. P. & Koslowsky, T. C. Percutaneous iliosacral screw fixation after osteoporotic posterior ring fractures of the pelvis reduces pain significantly in elderly patients. *Injury* **46,** 1631–1636 (2015).  13. Höch, A., Özkurtul, O., Pieroh, P., Josten, C. & Böhme, J. Outcome and 2-Year Survival Rate in Elderly Patients With Lateral Compression Fractures of the Pelvis. *Geriatr. Orthop. Surg. Rehabil.* **8,** 3–9 (2017).  14. Buller, L. T., Best, M. J. & Quinnan, S. M. A Nationwide Analysis of Pelvic Ring Fractures: Incidence and Trends in Treatment, Length of Stay, and Mortality. *Geriatr. Orthop. Surg. Rehabil.* **7,** 9–17 (2016).  15. Balogh, Z. *et al.* The epidemiology of pelvic ring fractures: a population-based study. *J. Trauma* **63,** 1066-73–3 (2007).  16. Kannus, P., Parkkari, J., Niemi, S. & Sievänen, H. Low-Trauma Pelvic Fractures in Elderly Finns in 1970-2013. *Calcif. Tissue Int.* **97,** 577–80 (2015).  17. Burge, R. *et al.* Incidence and Economic Burden of Osteoporosis-Related Fractures in the United States, 2005-2025. *J. Bone Miner. Res.* **22,** 465–475 (2007).  18. Hernlund, E. *et al.* Osteoporosis in the European Union: medical management, epidemiology and economic burden. A report prepared in collaboration with the International Osteoporosis Foundation (IOF) and the European Federation of Pharmaceutical Industry Associations (EFPIA). *Arch. Osteoporos.* **8,** 136 (2013). |

| **3. Wissenschaftliche Angaben (Fortsetzung):** |
| --- |
| *Detaillierte Zielsetzung:* |
| Die Studie soll Hinweise mit adäquatem Evidenzlevel zur Therapie der oben genannten Fraktur liefern und die Lebensqualität der betroffenen wie auch zukünftigen Patienten verbessern.  Ferner sollen die erhobenen Daten zur Entwicklung eines neuen evidenzbasierten Behandlungsalgorithmus sowie zur wissenschaftlichen Verwertung und Publikation dienen.  Der klinische Behandlungspfad am Uniklinikum Hamburg-Eppendorf im Fall von Frakturen des hinteren Beckenrings wurde anhand der vorhandenen Studien und nach bestem klinischen Wissen modifiziert. Ziel ist es durch die Randomisierung eine direkte Kontrolle der operativen Therapie zu ermöglichen, schlussendlich Hinweise auf hohem Evidenzniveau zu sammeln und einen optimalen Behandlungsalgorithmus entwickeln zu können. |
| *Relevanz:* |
| Die Bedeutung der hier untersuchten Verletzung ist für unsere alternde Gesellschaft und medizinisch-traumatologische Ärzteschaft immens.  Die Angaben zur Häufigkeit in der Literatur schwanken zwischen 23-92 Insuffizienzfrakturen pro 100.000 Einwohner pro Jahr.^14–16^ Vor kurzem veröffentlichte Studien zeigen eine deutliche Zunahme dieser Fraktur-Entität.  In Finnland beispielsweise kam es von 1970 bis 1997 zu einem Anstieg von 470% von osteoporotischen Frakturen des Beckens. Insgesamt sind 7% aller osteoporotisch bedingten Frakturen dem Beckenring zuzuordnen^16,17^. Übertragen auf die Population in der Europäischen Union, deren Einwohner älter als 50 Jahre bis 2025 um 20% zunehmen und Einwohner älter als 80 Jahre um 32%^18^ zunehmen werden, heißt dies, das im Jahr 2025 ca. 34 Millionen Menschen von einer osteoporotischen Fraktur betroffen sein werden. Folgerichtig würde dies das Vorkommen von ca. 2,38 Millionen Insuffizienz-Frakturen des hinteren Beckenrings in der EU in 2025 bedeuten. Da Frakturen des hinteren Beckenrings mit osteoporotischen Veränderungen des Knochens zusammenhängen^2^ und der demographische Wandel fortschreitet wird diese Art von Verletzung die Unfallchirurgie vor eine große Herausforderung stellen. |
| *Methoden (Material, Messtechnik, Statistik, etc.):* |
| Alle Patienten über 60 Jahre mit einer hinteren Beckenringfraktur Typ B2.1 oder FFP II oder unter 60 Jahre mit einer osteoporotisch oder osteopen bedingten hinteren Beckenringfraktur des gleichen Typs, welche am Universitätsklinikum Hamburg-Eppendorf aufgenommen und versorgt werden, sollen prospektiv erfasst und randomisiert werden, sofern eine entsprechende Einwilligungserklärung des Patienten oder gesetzlichen Betreuers vorliegt.  Falls der/die Patient/in keine gesetzliche Betreuung hat, aber klinisch nicht mehr Einwilligungsfähig erscheint, wird eine Eilbetreuung binnen 24h eingerichtet. Falls dies ebenfalls nicht gelingt, wird der/die Patientin nicht in die Studie eingeschlossen.  Die Randomisierung wird am Aufnahmetag, spätestens am ersten stationären Tag durch einen der Prüfärzte vorgenommen. Zuvor werden 130 Umschläge (OP oder konservativ) in einem abschließbaren Behälter gefüllt und zufällig vermengt.  Bei beiden Therapiearmen wird jeweils auf klinisch bewährte Behandlungsregimes zurückgegriffen.  Gruppe I: Es wird innerhalb der ersten 1-3 Tage eine operative perkutan-minimalinvasive Versorgung mittels einer transiliosakralen Schraubenosteosynthese, sowie bei bestehender Indikation Stabilisation des vorderen Beckenringes (üblicherweise: Anlage eines supraacetabulären Fixateur externes) durchgeführt. Am ersten postoperativen Tag wird die Mobilisation mithilfe der Physiotherapie erfolgen. Am zweiten postoperativen Tag wird durch Röntgen und Kontroll-CT die Schraubenfehllage ausgeschlossen.  Eine erste Nachuntersuchung wird nach 6 Wochen in unserer Poliklinik erfolgen. Es werden Fragebögen nach Barthel, Tinetti sowie der VAS Score erhoben.  Gruppe II: Es wird eine analgetische Abdeckung nach dem WHO Stufenschema und eine Mobilisation nach 1-3 Tagen mithilfe der Physiotherapie durchgeführt. Vor Entlassung und nach Belastung wird eine Röntgen-Verlaufskontrolle zum Ausschluss einer groben Dislokation stattfinden. Eine erste klinisch-CT graphische Nachuntersuchung wird nach 2 Wochen in unserer Poliklinik erfolgen, falls hier bislang keine Mobilisation möglich war oder weiterhin starke Schmerzen (VAS >5) vorliegen wird der/die Teilnehmer/in in eine dritte Gruppe (III = verzögerte operative Therapie) eingeschlossen. Es wird dann eine operative Therapie nach den oben beschriebenen Maßgaben initiiert, der weitere Ablauf ist entsprechend Gruppe I.  Die zweite Untersuchung der Gruppe II wird nach 6 Wochen stattfinden. Es werden Fragebögen nach Barthel, Tinetti sowie der VAS Score erhoben.  Für alle Gruppen sind dann Nachuntersuchungen nach 6, 12 und 24 Monaten mit erneuter Erhebung der Scores geplant. |
| *Erwartete Ergebnisse (Hypothese):* |
| Aufgrund einschlägiger klinischer Erfahrungen wird angenommen, dass der konservative Arm dem operativen langfristig nicht unterlegen sein wird, jedoch der operative Arm einen kurzfristigen positiven analgetischen Effekt aufweist und damit eine Frühmobilisation ermöglicht, welche im geriatrischen Krankengut bei anderen Verletzungen der unteren Extremität Überlebensvorteile ergab.  Somit gilt als Hypothese, dass die operative minimalinvasive perkutane Schraubenosteosynthese die Liegedauer, die Dauer und Intensität der Schmerzen und die damit verbundene Morbidität und Mortalität im Vergleich zur konservativen Therapie senken wird. Ferner wird von einer Verbesserung der Lebensqualität nach operativer Versorgung ausgegangen. |

Mit freundlichen Grüßen

PD Dr. med. Maximilian Hartel Dr. med. Darius Thiesen

Oberarzt, Klinik für Unfallchirurgie Assistenzarzt, Klinik für Unfallchirurgie

Universitätsklinik Hamburg-Eppendorf Universitätsklinik Hamburg-Eppendorf
